# Effect of COVID-19 pandemic on outcomes in intracerebral hemorrhage

**DOI:** 10.1371/journal.pone.0284845

**Published:** 2023-04-26

**Authors:** Daryl C. McHugh, Anna Gershteyn, Christine Boerman, Robert G. Holloway, Debra E. Roberts, Benjamin P. George

**Affiliations:** Department of Neurology, University of Rochester Medical Center, Rochester, NY, United States of America; Foshan Sanshui District People’s Hospital, CHINA

## Abstract

**Objectives:**

Patients with severe intracerebral hemorrhage (ICH) often suffer from impaired capacity and rely on surrogates for decision-making. Restrictions on visitors within healthcare facilities during the pandemic may have impacted care and disposition for patient with ICH. We investigated outcomes of ICH patients during the COVID-19 pandemic compared to a pre-pandemic period.

**Materials and methods:**

We conducted a retrospective review of ICH patients from two sources: (1) University of Rochester Get With the Guidelines database and (2) the California State Inpatient Database (SID). Patients were divided into 2019–2020 pre-pandemic and 2020 pandemic groups. We compared mortality, discharge, and comfort care/hospice. Using single-center data, we compared 30-day readmissions and follow-up functional status.

**Results:**

The single-center cohort included 230 patients (n = 122 pre-pandemic, n = 108 pandemic group), and the California SID included 17,534 patients (n = 10,537 pre-pandemic, n = 6,997 pandemic group). Inpatient mortality was no different before or during the pandemic in either cohort. Length of stay was unchanged. During the pandemic, more patients were discharged to hospice in the California SID (8.4% vs. 5.9%, p<0.001). Use of comfort care was similar before and during the pandemic in the single center data. Survivors in both datasets were more likely to be discharged home vs. facility during the pandemic. Thirty-day readmissions and follow-up functional status in the single-center cohort were similar between groups.

**Conclusions:**

Using a large database, we identified more ICH patients discharged to hospice during the COVID-19 pandemic and, among survivors, more patients were discharged home rather than healthcare facility discharge during the pandemic.

## Introduction

The COVID-19 pandemic placed a strain on hospital resources, impacted access to acute stroke therapies, and reduced patient visitation to healthcare facilities, including hospitals and nursing homes, for families and surrogate decision-makers based on widely utilized policies intended to prevent the transmission of COVID-19.

In-hospital mortality rates for intracerebral hemorrhage (ICH) range from 20–30%, and long-term functional dependence is 50% or more [1]. Patients admitted with severe ICH often suffer from functional and cognitive deficits and may rely on surrogates for advance care and discharge planning, including Do-Not-Resuscitate (DNR), tracheostomy or gastrostomy placement, hospice or comfort measures. Patient preferences regarding life-sustaining therapy are documented in less than 40% of dying stroke patients, and most inpatient deaths occur after transitioning to comfort measures only (CMO) [2, 3]. ICH patients may have been disproportionately affected by hospital visitor restrictions and limitations in surrogate communication during the COVID-19 pandemic.

The impact of the COVID-19 pandemic on decision-making, discharge planning, and outcomes in acute ICH patients is not well described. We sought to characterize outcomes in patients admitted with acute ICH during a period of the COVID-19 pandemic (compared to a pre-pandemic time-period) from a single institution in New York and a statewide database from California.

## Materials and methods

We conducted analyses of two separate databases: (1) single-center American Heart Association/American Stroke Association Get With The Guidelines (GWTG) Stroke data, and (2) California State Inpatient Database (CA_SID). Additional methodology details are available in the **S1 Appendix**.

### Single-center GWTG database

We conducted a retrospective observational study of adult (age ≥18 years) patients admitted with a primary diagnosis of ICH from June 1, 2019 to December 31, 2020 to the University of Rochester Medical Center in Rochester, New York. Patients were identified by ICH diagnosis in the GWTG database and reviewed in the electronic medical record for accuracy. Patients were included if ICH was due to hypertension, cerebral amyloid angiopathy, vascular malformations, or cryptogenic. Exclusions were made for ischemic stroke with hemorrhagic transformation, subarachnoid hemorrhage, subdural hemorrhage, and hemorrhagic metastatic lesions. Patients were divided into a pre-pandemic group with admission from June 1, 2019 to March 17, 2020, and a pandemic group with admission from March 18, 2020 to December 31, 2020. We recorded demographics, baseline modified Rankin Scale (mRS), ICH score, pre-existing advance directives, and Healthcare Proxy status and relationship.

Outcomes analyzed included hospital and intensive care unit (ICU) length of stay, discharge disposition, discharge and follow-up mRS, transition to CMO, tracheostomy and gastrostomy placement, and 30-day hospital readmissions. All data were obtained directly from the GWTG database or in review of the electronic medical record. The study was approved by the University of Rochester Institutional Review Board with a waiver of informed consent.

### California State Inpatient Database

We conducted a retrospective observational study of adult (age ≥18 years) patients admitted with a primary diagnosis of ICH from January 1, 2019 to December 31, 2020 to acute care hospitals in the California. The CA_SID includes a complete enumeration of all-payer administrative claims data on hospital discharges from all non-federal acute care hospitals within California in each year. Patients were identified using the International Classification of Disease 10^th^ Revision (ICD-10) code I61 for nontraumatic intracerebral hemorrhage. Exclusions were made for hospitalizations with comorbid traumatic brain injury (ICD-10 codes S0[247], S06[12345689]), comorbid brain tumor (ICD-10 C71, C7931), elective admission status, and missing data for patient covariates or outcomes.

Patients were divided into a pre-pandemic group (admission from January 1, 2019 to February 28, 2020) and a pandemic group (admission from March 1, 2020 to December 31, 2020). We recorded patient demographics, median household income for patient’s ZIP code, and insurance payer. Elixhauser comorbidity index was also calculated. Procedures codes were used to identify patients that underwent invasive mechanical ventilation, cranial neurosurgery, and ventriculostomy.

Outcomes analyzed included early DNR status (defined as DNR order placed within 24 hours of admission), tracheostomy and gastrostomy, discharge disposition, inpatient death, days from admission to tracheostomy/gastrostomy, survivor length of stay, and time from admission to death.

### Statistical analysis

Single-center data were analyzed descriptively. Chi-squared and Wilcoxon-Rank Sum tests were used for categorical and continuous variables, respectively. For analyses of the CA_SID, multivariable models adjusting for known patient covariates were used to assess outcomes before and during the pandemic. Logistic regression was used for dichotomous outcomes, negative binomial regression was used for count data (e.g., length of stay), and Cox-Proportional Hazard models were used for mortality. Cluster robust variance estimators were used to account for patient clustering within hospital. Two-sided significance level of p<0.05 was set *a priori*. Statistical analyses were performed using STATA statistical software version 17.0 (StataCorp, College Station, TX) [4].

## Results

### Single-center GWTG analysis

Among 238 adult patients identified in the timeframe, exclusions were made for ischemic stroke with hemorrhagic transformation (n = 4), subarachnoid hemorrhage (n = 1), cerebral venous sinus thrombosis (n = 2), and hemorrhagic metastasis (n = 1). There were 122 ICH patients in the pre-pandemic period and 108 ICH patients in pandemic period. Baseline characteristics between groups were similar (**Table 1**). Patients admitted during the pandemic were more likely to have a poor baseline functional status (mRS 3–5 (moderate to severe functional disability) vs. mRS 0–2 (no to slight functional disability), p = 0.012). Most patients had no advanced directives and approximately half of all patients had an assigned Healthcare Proxy on admission.

**Table 1:** Patient characteristics in the single-center get with the guidelines data.

Patient Characteristic	Pre-Pandemic^a^	Pandemic^a^	P value
Female, n (%)	53 (43)	48 (44)	0.98
Race and Ethnicity, n (%)			
Asian	5 (4)	6 (6)	0.94
Black	17 (14)	14 (13)	
Hispanic	2 (2)	2 (2)	
Non-Hispanic White	97 (80)	84 (78)	
Other^b^	1 (1)	2 (2)	
Age, years, mean (SD)	69.6 (14.7)	67.7 (15.1)	0.34
Baseline mRS^c^, n (%)			
0–2	90 (87)	73 (73)	0.012
3–5	13 (13)	27 (27)	
ICH Score, n (%)			
0	30 (28)	22 (22)	0.27
1	22 (21)	25 (25)	
2	18 (17)	22 (22)	
3	19 (18)	24 (24)	
4	7 (7)	5 (5)	
5	10 (9)	3 (3)	
Pre-Morbid Advanced Directives, n (%)			
None	88 (83)	84 (83)	0.62
DNR Only	11 (10)	13 (13)	
DNR/DNI	7 (7)	4 (4)	
Assigned HCP, n (%)	55 (53)	45 (45)	0.29
HCP Relation, n (%)			
Spouse	19 (35)	18 (40)	0.26
Child	23 (42)	20 (44)	
Sibling	6 (11)	0 (0)	
Parent	2 (4)	4 (9)	
Other^d^	5 (9)	3 (6)	

Abbreviations: SD = Standard Deviation; mRS = modified Rankin Scale; ICH = Intracerebral Hemorrhage; DNR = Do-Not-Resuscitate; DNI = Do-Not-Intubate; HCP = Health Care Proxy

^a^ The pre-pandemic period is from June 1, 2019 to February 29, 2020 and the pandemic period is March 1, 2020 to December 31, 2020.

^b^ Includes American Indian/Alaskan Native and unknown

^c^ Missing data for 19 patients in the pre-pandemic period and 8 patients during the pandemic period.

^d^ Other health care proxy include friend (n = 5), sibling-in-law (n = 2), niece (n = 1).

Outcomes assessed before and during the pandemic were similar for ICH patients (**Table 2**). Inpatient mortality was similar between groups with n = 42 (43%) deaths before the pandemic compared to n = 33 (31%) deaths during the pandemic (p = 0.39). Survivors were more likely to be discharged home instead of rehabilitation or skilled nursing facility during the pandemic (n = 37 (49%) vs. n = 26 (33%), p = 0.033). Discharge mRS, CMO and timing of the CMO were not significantly different between groups. Few patients underwent tracheostomy and/or gastrostomy. Follow-up data were available for n = 60 (70%) of survivors in the pre-pandemic group and n = 58 (74%) of survivors in the pandemic group. The were no difference in follow-up mRS or readmission among survivors in each group.

**Table 2:** Patient outcomes in the single-center get with the guidelines data.

Outcomes	Pre-Pandemic^a^	Pandemic^a^	p value
Total	122 (100)	108 (100)	NA
Hospital Length of Stay, days, mean (SD)	11.0 (12.7)	11.8 (20.0)	0.73
ICU Length of Stay, days, mean (SD)	5.2 (7.4)	5.1 (12.0)	0.97
Disposition, n (%)			
Home	26 (21)	37 (34)	0.23
Acute Rehabilitation	27 (22)	17 (16)	
SNF	27 (22)	21 (19)	
Hospice	6 (5)	3 (3)	
Death	36 (30)	30 (28)	
Survivor Disposition, n (%)			
Home	26 (33)	37 (49)	0.033
Acute Rehabilitation or SNF	54 (68)	38 (51)	
Discharge mRS^b^, n (%)			
0–2	11 (9)	14 (13)	0.35
3–6	110 (91)	94 (87)	
Follow-up mRS^c^, n (%)			
0–2	31 (52)	26 (45)	0.46
3–6	29 (48)	32 (55)	
CMO, n (%)	38 (31)	34 (31)	1.0
Time to CMO, n (%)			
Within 48 hours	16 (42)	15 (44)	1.0
>48 hours from admission	22 (58)	19 (56)	
Time to CMO Decision, days, mean (SD)	6.6 (9.2)	7.7 (19.8)	0.75
Tracheostomy, n (%)	3 (2)	3 (7)	1.0
PEG, n (%)	7 (16)	10 (22)	0.66
Time to Tracheostomy/PEG Decision, days, mean (SD)	15 (5.2)	13.3 (5.7)	0.52

Abbreviations: SD = Standard Deviation; ICU = Intensive Care Unit; SNF = Skilled Nursing Facility; mRS = modified Rankin Scale; CMO = Comfort Measures Only; PEG = Percutaneous Endoscopic Gastrostomy

^a^ The pre-pandemic period is from June 1, 2019 to February 29, 2020 and the pandemic period is March 1, 2020 to December 31, 2020.

^b^ Missing data for one patient in the pre-pandemic period.

^c^ Median time to follow-up in the pre-pandemic group was 118 days (IQR 38.5–423) and 150 days (IQR 67–354) in the pandemic group. Data is restricted to patients who survived hospital discharge and followed in clinic or were re-admitted (n = 60 (70%) in the pre-pandemic group and n = 58 (74%) in the pandemic group).

### California State Inpatient Database analysis

Among 19,577 adult ICH patients identified in the timeframe, exclusions were made for hospitalizations with comorbid traumatic brain injury (n = 145), comorbid brain tumor (n = 536), elective admission status (n = 369), and missing data for patient covariates or outcomes (n = 994). There were 10,537 ICH patients in the pre-pandemic period and 6,997 ICH patients in the pandemic period admitted to acute care hospitals in California. Patients admitted during the pandemic period were older and more often insured with Medicaid. Patients identified as Black or Non-Hispanic White were admitted with ICH less during the pandemic compared to the pre-pandemic period (**Table 3**).

**Table 3:** Patient characteristics for ICH admissions in the California State Inpatient Database.

Patient Characteristic	Pre-Pandemic^a^		Pandemic^a^		P value
Total, n (%)	10,537	(100)	6,997	(100)	NA
Age in years, mean (SD)	66.8	(15.8)	65.9	(15.9)	<0.001
Female, n (%)	4,728	(44.9)	3,039	(43.4)	0.06
Race and ethnicity, n (%)					
Asian or Pacific Islander	2,034	(19.3)	1,390	(19.9)	<0.001
Black	961	(9.1)	531	(7.6)	
Hispanic	2,901	(27.5)	2,083	(29.8)	
Non-Hispanic White	4,064	(38.6)	2,532	(36.2)	
Other^b^	577	(5.5)	461	(6.6)	
Median household income for ZIP^c^, n (%)					
1st quartile	2,896	(27.5)	1,947	(27.8)	0.73
2nd quartile	2,684	(25.5)	1,761	(25.2)	
3rd quartile	2,634	(25.0)	1,711	(24.5)	
4th quartile	2,323	(22.0)	1,578	(22.6)	
Insurance payer, n (%)					
Private	2,233	(21.2)	1,492	(21.3)	<0.001
Medicare	5,688	(54.0)	3,602	(51.5)	
Medicaid	2,147	(20.4)	1,612	(23.0)	
Self pay/No charge/Other^d^	469	(4.5)	291	(4.2)	
Elixhauser Index^e^, mean (SD)	4.2	(2.1)	4.3	(2.0)	0.004
Invasive Mechanical Ventilation	3,381	(32.1)	2,244	(32.1)	0.98
Cranial Neurosurgical Procedure	1,517	(14.4)	1,068	(15.3)	0.11
Ventriculostomy	1,171	(11.1)	814	(11.6)	0.29

Abbreviations: NA = Not Applicable; SD = Standard Deviation; ZIP = Zone Improvement Plan

^a^ The pre-pandemic period is from January 1, 2019 to February 29, 2020 and the pandemic period is March 1, 2020 to December 31, 2020.

^b^ “Other” race and ethnicity includes individuals not categorized by the California State Inpatient Database, which include those identified as multiple races, other race not classified, or unknown. Individuals identified as Native American and Alaskan Native are included within this group for confidentiality reasons due to fewer than 10 records within the sample.

^c^ Household income quartiles were assigned based on the median income of the patient’s ZIP Code, where the first quartile is the lowest income and fourth quartile is the highest income.

^d^ “Other” insurance payer includes self-pay, no charge, Worker’s Compensation, Civilian Health and Medical Program of the Uniformed Services or Veterans Affairs, Title V, and other government programs.

^e^ Elixhauser comorbidity index is a measure of comorbidity for use with large administrative datasets with higher numbers representing the presence of more comorbidities, accounting for up to 31 categories of disease.

**Fig 1:**
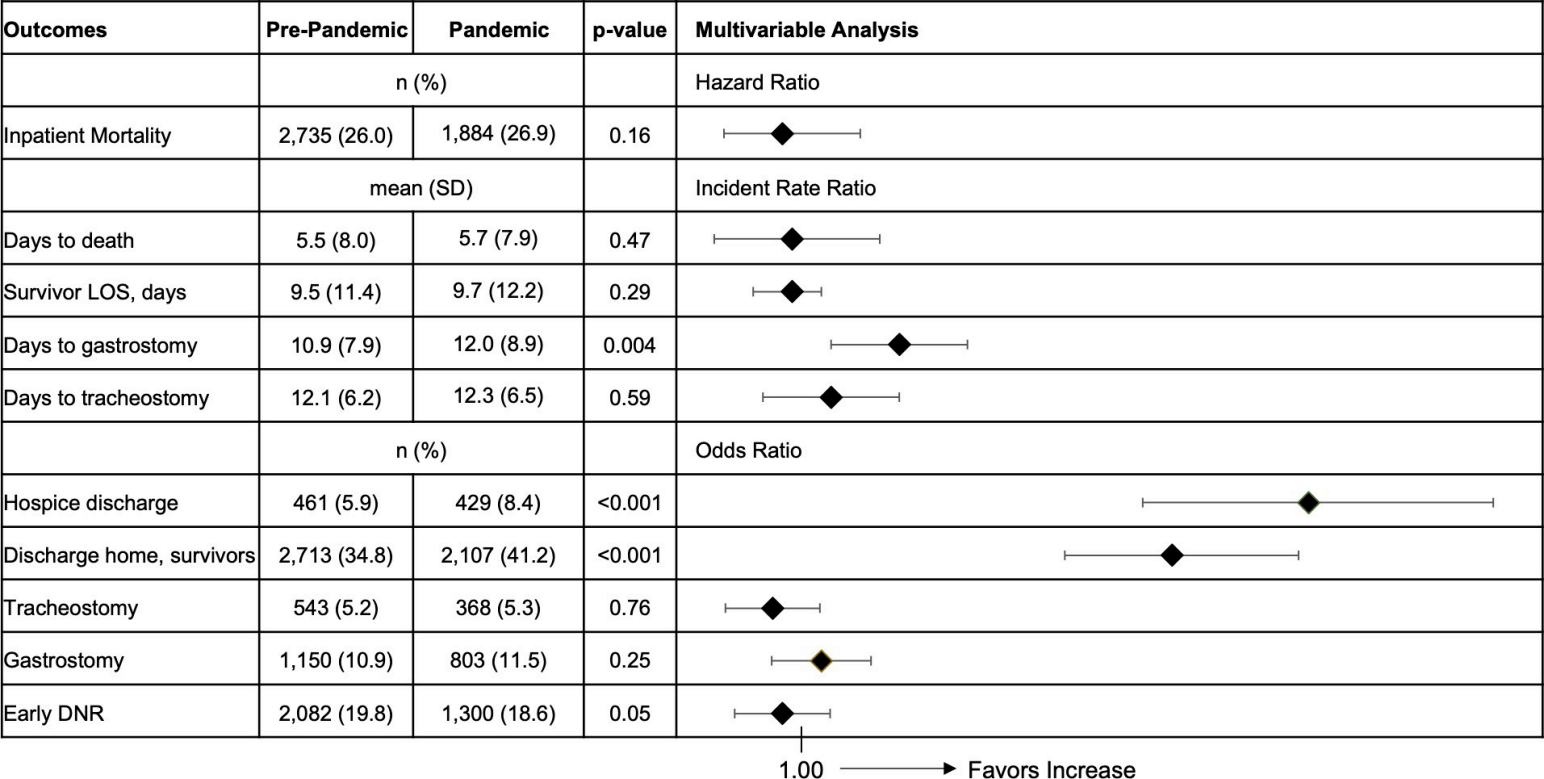
Patient outcomes for ICH admissions in the California State Inpatient Database. The pre-pandemic period is from January 1, 2019 to February 29, 2020 and the pandemic period is March 1, 2020 to December 31, 2020. Days to gastrostomy/tracheostomy is from time of admission. Hospice discharge includes hospice facility and home hospice. Early DNR is defined as DNR within 24 hours of admission. Multivariable analyses are adjusted for age, sex, race and ethnicity, median household income for patient ZIP, insurance payer, Elixhauser Comorbidity Index, mechanical ventilation, cranial neurosurgical procedure, and ventriculostomy.

Adjusted mortality, length of stay, and time awaiting tracheostomy was unchanged in the pandemic period. Time awaiting gastrostomy was slightly increased in the pandemic (Incident Rate Ratio 1.10 95% CI 1.03–1.18). The odds of tracheostomy, gastrostomy, and early DNR were unchanged comparing the pandemic to the pre-pandemic period. However, the adjusted odds of discharge to home (Odds Ratio 1.38 95% CI 1.27–1.51) and hospice (Odds Ratio 1.52 95% CI 1.35–1.71) increased during the pandemic compared to the pre-pandemic period (**Fig 1**).

## Discussion

We investigated outcomes during the COVID-19 pandemic of ICH patients using both single-center data with clinical detail and large, administrative data. In both datasets, compared to the pre-pandemic period, there was no significant difference in mortality or length of stay. In our single-center data, there was no difference in discharge mRS, frequency of transition to CMO, or time to CMO decision. During the pandemic period, survivors were more likely to be discharged home compared to acute rehabilitation or skilled nursing facility. Follow-up mRS and 30-day readmissions were no different between groups. In the CA_SID, frequency of tracheostomy, gastrostomy placement, and early DNR orders was similar between groups. The pandemic period demonstrated a longer time between admission and gastrostomy tube placement. During the pandemic period, there were fewer admissions among patients identifying as Black. Prior data has reported increased healthcare disparities amongst Black patients with acute stroke during the pandemic [5]. Patients were also more likely to be discharged to hospice during the pandemic, and among survivors, more likely to be discharged home vs. post-acute care facility.

While clinicians rely on surrogate decision-makers to help guide goal-concordant care for patients with severe brain injury, the COVID-19 pandemic divided providers and family members of patients due to hospital and medical facility visitation restrictions. Family members of patients admitted to ICUs during COVID-19 visitation restrictions reported increased difficulty comprehending the patient’s clinical status and that phone and video communications were inferior compared to in-person discussions [6]. Family members of brain injured patients had increased difficulty coping with grief and uncertainty, advocating for the patient, and building trust with the clinical team compared to prior to visitation restrictions [7]. Despite visitation restrictions, we did not observe changes in mortality, frequency of or time to CMO, early DNR orders, and frequency of tracheostomy and gastrostomy placement. There was a slight increase in time to gastrostomy placement in the CA_SID, although this may be secondary to factors delaying procedures during the pandemic (such as surgical staffing, equipment shortages, and precautions taken during the pandemic) rather than delays in goals of care decision-making.

The University of Rochester Medical Center neurointensive care team took efforts to promote communication with surrogate decision-makers during the period of visitation restriction. Surrogates received daily phone calls from clinicians to discuss patient events, progress, and care plans. Bedside nurses also provided updates and participated in video visits between patients and family members when requested. Separate goals of care meetings, which may have included multiple family members via phone or video conferences, were scheduled as needed. The attempts to increase alternative forms of communication between surrogates and the clinical team during this period may have also contributed to similar outcomes during the pandemic. Other variables, such as personal beliefs, values, and socioeconomic factors, may have a larger role in goals of care decision-making. Surrogates who elected to pursue tracheostomy placement for severe brain injury patients have reported they felt there was no appropriate alternative when prognostic uncertainty existed [8].

In the CA_SID, we observed an increase in discharge to hospice care for ICH patients. Potential reasons for this include reduced availability of skilled nursing facility beds during the pandemic and inability of caregivers to provide the level of care needed for sicker patients at home. Furthermore, during COVID surges, providers may have been more likely to engage in early and persuasive goals of care discussions with quicker triage of severe ICH cases especially given the high inpatient and ICU demands from COVID-related respiratory failure [9]. Withdrawal of life-sustaining therapy occurs in up to 76% of patients dying from ICH, and predicted mortality by ICH score is likely impacted by withdrawal of life-sustaining therapy [10–12]. The Neurocritical Care Society recommends determining prognosis from repeated evaluations over time, with at least a 72 hour observation period, to determine clinical response and establish greater confidence and accuracy in prognosis [13]. Early goals of care discussions may have contributed to an increase in hospice discharges in patients who may have had acceptable functional outcomes if a more prolonged course of acute therapy were pursued.

Although we did not specifically investigate outcomes of ICH patients with COVID-19, increased mortality and worse functional outcomes have been reported in patients presenting with acute ischemic stroke and concurrent COVID-19 infection [14]. In addition, while overall ICH admissions were decreased during the pandemic, more ICH patients were admitted to low-volume ICH centers than high-volume ICH centers during the pandemic [15]. This may have been a result of bed capacity limitations at high-volume centers due to increased COVID-related admissions. The shift in ICH admissions towards low-volume centers may have also impacted outcomes and goals of care conversations during this period.

During the pandemic, significantly more ICH survivors were discharged home in both datasets. Limited data exists regarding discharge destination for acute stroke patients during the pandemic, with mixed findings [16, 17]. Potential factors contributing to the increase in home discharges may include patient preference (e.g. avoidance of COVID-19 exposure at inpatient facilities), limited bed availability at inpatient facilities, and increased availability of caregiver support at home with shelter-in-place orders and more people working from home.

The increase in home discharges during the pandemic did not have a negative impact on outcomes as 30-day readmission rates and follow-up mRS in our single-center sample were similar between groups. Our findings suggest that more ICH patients may potentially be discharged directly home without negatively impacting mortality or long-term functional outcomes. An increase in home discharges could increase patient and family satisfaction as well as reduce healthcare costs.

### Limitations

Our study is limited by its retrospective design and limitation to ICH patients, limiting the generalizability to the neurocritical care population as a whole. The single-center dataset was small and predominantly White race. The CA_SID is an administrative database with diagnoses based upon ICD-10 codes. While coding errors may exist, the sensitivity and positive predictive value of utilizing ICD-10 codes for identifying acute hemorrhagic strokes is 99% and 89%, respectively [18]. Patients in this sample were selected based upon admission date, so individual admitted in December 2020 but discharged after December 2020 were not included, leading to a loss of some patients. Functional outcome data were not available in the CA_SID. There is some data to suggest that ICH rates and outcomes may vary between seasons [19]. As our pre-pandemic and pandemic groups do not correspond to identical seasons during the time periods included, differences in ICH rates and outcomes due to seasonal variations cannot be accounted for.

## Conclusions

We did not observe a difference in frequency of CMO, DNR, tracheostomy and gastrostomy placement for ICH patients during the pandemic, but there was a significant increase in discharge to hospice. Furthermore, more patients were discharged home during the pandemic without an increase in 30-day readmissions or decline in follow-up functional status in our single-center cohort. It may be possible to consider greater home discharges among candidate ICH patients without negatively impacting outcomes.

## Supporting information

S1 Appendix Table A.1: STROBE Checklist. Table A.2: Additional Methodology Details. Table A.3: Comorbidity Codes.
(DOCX)
