# Supplementary material for: Effect of COVID-19 pandemic on outcomes in intracerebral hemorrhage
Source: PLoS One. 2023 Apr 26;18(4):e0284845. doi: 10.1371/journal.pone.0284845 (PMC10132587; doi:10.1371/journal.pone.0284845)
Supplement: S1 Appendix — Table A.1: STROBE Checklist. Table A.2: Additional Methodology Details. Table A.3: Comorbidity Codes. (DOCX) [file pone.0284845.s001.docx]

**Appendix A**

**Effect of COVID-19 Pandemic on Outcomes in Intracerebral Hemorrhage**

**Table of Contents**

| **Title** | **Page #** |
| --- | --- |
| Table A.1: STROBE Checklist …………………………………………………………….. | 1 |
| Table A.2: Additional Methodology Details ………………………………………………... | 3 |
| Table A.3: Comorbidity Codes ……………………………………………………………… | 4 |

**Table A.1:** STROBE Checklist

|  | Item No | Recommendation | Page  No |
| --- | --- | --- | --- |
| **Title and abstract** | 1 | (*a*) Indicate the study’s design with a commonly used term in the title or the abstract | 2 |
|  |  | (*b*) Provide in the abstract an informative and balanced summary of what was done and what was found | 2 |
| Introduction | | | |
| Background/rationale | 2 | Explain the scientific background and rationale for the investigation being reported | 3 |
| Objectives | 3 | State specific objectives, including any prespecified hypotheses | 3 |
| Methods | | | |
| Study design | 4 | Present key elements of study design early in the paper | 4 |
| Setting | 5 | Describe the setting, locations, and relevant dates, including periods of recruitment, exposure, follow-up, and data collection | 4 |
| Participants | 6 | (*a*) *Cohort study*—Give the eligibility criteria, and the sources and methods of selection of participants. Describe methods of follow-up  *Case-control study*—Give the eligibility criteria, and the sources and methods of case ascertainment and control selection. Give the rationale for the choice of cases and controls  *Cross-sectional study*—Give the eligibility criteria, and the sources and methods of selection of participants | 4 |
|  |  | (*b*) *Cohort study*—For matched studies, give matching criteria and number of exposed and unexposed  *Case-control study*—For matched studies, give matching criteria and the number of controls per case | N/A |
| Variables | 7 | Clearly define all outcomes, exposures, predictors, potential confounders, and effect modifiers. Give diagnostic criteria, if applicable | 4 |
| Data sources/ measurement | 8* | For each variable of interest, give sources of data and details of methods of assessment (measurement). Describe comparability of assessment methods if there is more than one group | *4* |
| Bias | 9 | Describe any efforts to address potential sources of bias | N/A |
| Study size | 10 | Explain how the study size was arrived at | N/A |
| Quantitative variables | 11 | Explain how quantitative variables were handled in the analyses. If applicable, describe which groupings were chosen and why | 5 |
| Statistical methods | 12 | (*a*) Describe all statistical methods, including those used to control for confounding | 5 |
|  |  | (*b*) Describe any methods used to examine subgroups and interactions | 5 |
|  |  | (*c*) Explain how missing data were addressed | Supplement |
|  |  | (*d*) *Cohort study*—If applicable, explain how loss to follow-up was addressed  *Case-control study*—If applicable, explain how matching of cases and controls was addressed  *Cross-sectional study*—If applicable, describe analytical methods taking account of sampling strategy | N/A |
|  |  | (*e*) Describe any sensitivity analyses | N/A |

Continued on next page

| Results | | | |
| --- | --- | --- | --- |
| Participants | 13* | (a) Report numbers of individuals at each stage of study—eg numbers potentially eligible, examined for eligibility, confirmed eligible, included in the study, completing follow-up, and analysed | 6, 8 |
|  |  | (b) Give reasons for non-participation at each stage | N/A |
|  |  | (c) Consider use of a flow diagram | N/A |
| Descriptive data | 14* | (a) Give characteristics of study participants (eg demographic, clinical, social) and information on exposures and potential confounders | Tables 1 & 3 |
|  |  | (b) Indicate number of participants with missing data for each variable of interest | Tables 1 & 3 |
|  |  | (c) *Cohort study*—Summarise follow-up time (eg, average and total amount) | Table 2 |
| Outcome data | 15* | *Cohort study*—Report numbers of outcome events or summary measures over time | 6, 8 |
|  |  | *Case-control study—*Report numbers in each exposure category, or summary measures of exposure | *N/A* |
|  |  | *Cross-sectional study—*Report numbers of outcome events or summary measures | *N/A* |
| Main results | 16 | (*a*) Give unadjusted estimates and, if applicable, confounder-adjusted estimates and their precision (eg, 95% confidence interval). Make clear which confounders were adjusted for and why they were included | N/A |
|  |  | (*b*) Report category boundaries when continuous variables were categorized | Tables 1-4 |
|  |  | (*c*) If relevant, consider translating estimates of relative risk into absolute risk for a meaningful time period | N/A |
| Other analyses | 17 | Report other analyses done—eg analyses of subgroups and interactions, and sensitivity analyses | N/A |
| Discussion | | | |
| Key results | 18 | Summarise key results with reference to study objectives | 11 |
| Limitations | 19 | Discuss limitations of the study, taking into account sources of potential bias or imprecision. Discuss both direction and magnitude of any potential bias | 14 |
| Interpretation | 20 | Give a cautious overall interpretation of results considering objectives, limitations, multiplicity of analyses, results from similar studies, and other relevant evidence | 13 |
| Generalisability | 21 | Discuss the generalisability (external validity) of the study results | 13 |
| Other information | | | |
| Funding | 22 | Give the source of funding and the role of the funders for the present study and, if applicable, for the original study on which the present article is based | N/A |

**Table A.2:** Additional Methodology Details

| **American Heart Association/American Stroke Association Get With the Guidelines Stroke Database** | The GWTG-Stroke database is maintained at the University of Rochester Medical Center Strong Memorial Hospital, an 886 bed hospital and Joint Commission Certified Comprehensive Stroke Center. |
| --- | --- |
|  | To maintain consistency with the California SID, reporting of ethnicity was prioritized over race (e.g. patients documented as Black and Hispanic were recorded as Hispanic). |
|  | When the modified Rankin scale was not directly documented, estimates were produced based upon electronic medical record notes. |
|  | The California SID is limited to admission month, and day of admission cannot be identified in the database. |
|  | Race and ethnicity in California SID are directly reported by HCUP partner organizations and consolidated by HCUP to uniform values which combined race and ethnicity into a single variable. In HCUP methodology, if a patient was identified as Black and Hispanic, they were assigned to Hispanic. Additionally, HCUP consolidates some race categories (i.e., Asian and Native Hawaiian or Pacific Islander). |
|  | We used up to 36 secondary diagnoses to calculate the Elixhauser comorbidity index was also calculated from up to 31 categories of disease for each admission with the v2021.1 AHRQ Elixhauser Comorbidity Software (see Table A3). |
|  | Outcomes analyzed included early DNR status (defined as DNR order placed within 24 hours of admission), tracheostomy (ICD-10 procedure codes 0B11[034]F4, 0BH13EZ) and gastrostomy (ICD-10 procedure codes 0D16..4, 0DH[6A][03478]UZ) |

Abbreviations: GWTG-Stroke = Get With The Guidelines-Stroke; ICH = Intracerebral Hemorrhage; SID = State Inpatient Database; HCUP = Healthcare Cost and Utilization Project; DNR = Do Not Resuscitate; ICD-10 = International Classification of Diseases, Tenth Revision, Clinical Modification

**Table A.3:** Comorbidity Codes

| **Comorbidity Description** | **ICD-10-CM Diagnosis Codes (*regular expression*)^a^** |
| --- | --- |
| **Elixhauser Comorbidities^b^** |  |
| Congestive Heart Failure | I43, I50, I099, I110, I130, I132, I255, I42[056789], P290 |
| Cardiac Arrythmias | I44[123], I45[69], R00[018], T821, Z[49]50, I4[789] |
| Valvular Disease | A520, I09[18], Q23[012], Q23, Z95[234], I0[5678], I3[456789] |
| Pulmonary Circulation Disorders | I2[67], I28[089] |
| Peripheral Vascular Disorders | I7[01], I73[189], I771, I79[02], K55[189], Z95[89] |
| Hypertension, Uncomplicated^c^ | I10 |
| Hypertension, Complicated^c^ | I1[1235] |
| Paralysis | G041, G114, G80[12], G83[012349], G8[12] |
| Other Neurological Disorders | G25[45], G31[289], G93[14], R470, G1[0123], G2[012], G3[2567], G4[01], R56 |
| Chronic Pulmonary Disease | I27[89], J684, J70[13], J4[01234567], J6[01234567] |
| Diabetes, Uncomplicated^c^ | E1[0134][019], E120, E1[01234][2345678] |
| Diabetes, Complicated^c^ | E1[01234][2345678] |
| Hypothyroidism | E0[0123], E890 |
| Renal Failure | I120, I131, N250, Z49[012], Z940, Z992, N1[89] |
| Liver Disease | I864, I982, K71[13457], K76[023456789], Z944, K7[0234], B18, I85 |
| Peptic Ulcer Disease, Excluding Bleeding | K2[5678][79] |
| AIDS/HIV | B2[0124] |
| Lymphoma | C8[123458], C96, C90[02] |
| Metastatic Cancer | C7[789], C80 |
| Solid Tumor Without Metastasis | C[016][0123456789], C2[0123456], C3[01234789], C4[01356789], C5[012345678], C7[0123456], C97 |
| Rheumatoid Arthritis/Collagen Vascular Disease | M0[568], M3[02345], M45, L94[013], M12[03], M31[0123], M46[189] |
| Coagulopathy | D6[5678], D69[13456] |
| Obesity | E66 |
| Weight Loss | E4[0123456], R64, R634 |
| Fluid and Electrolyte Disorders | E8[67], E222 |
| Blood Loss Anemia | D500 |
| Deficiency Anemia | D50[89], D5[123] |
| Alcohol Abuse | G621, I426, K292, K70[039], Z502, Z714, Z721, F10, E52, T51 |
| Drug Abuse | F1[12345689], Z715, Z722 |
| Psychoses | F2[0234589], F3[01]2, F315 |
| Depression | F3[23], F204, F31[345], F341, F4[13]2 |
| **Other Comorbidity** |  |
| Dementia^d^ | F[0123456789][0123456789] |

Abbreviation: ICD-10-CM = International Classification of Diseases, Tenth Revision, Clinical Modification

a) Regular expression syntax allows for an abbreviation of several ICD-10 codes into a single term. The characters inside the brackets match a single alternating character for each separate code. A period represents a wildcard that serves as any character. For example, ABC..[AC] = ABCDFA, ABCDFC, ABCGHA. ABCGHC.

b) There are 36 equally weighted comorbidities that comprise the previously developed and validated Elixhauser Comorbidity Index.

c) Hypertension and Diabetes categories were consolidated for reporting purposes in Table 1.

d) Dementia was reported separately and is not part of the Elixhauser Comorbidity Index.
